# Modelling immunity gaps to quantify infection resurgences

**DOI:** 10.1098/rsos.250030

**Published:** 2025-07-16

**Authors:** Alex James, Reuben McGregor, Natalie Lorenz, Nicole J. Moreland, Miguel Moyers-Gonzalez

**Affiliations:** ^1^ University of Canterbury, Christchurch, Canterbury, New Zealand; ^2^ Te Punaha Matatini, Auckland, New Zealand; ^3^ Department of Molecular Medicine and Pathology, The University of Auckland, Auckland, New Zealand; ^4^ Maurice Wilkins Centre for Molecular Biodiscovery, Auckland, New Zealand

**Keywords:** disease resurgence, partial differential equation model, mathematical epidemiology

## Abstract

When COVID-19 restrictions were removed, many countries observed infection surges in respiratory pathogens like respiratory syncytial virus (RSV) and influenza. This has been postulated to have been caused by reduced immunity in populations due to non-pharmaceutical interventions that reduced transmission of these pathogens. This pandemic-related phenomenon has been termed ‘immunity debt’ or ‘immunity gap’. We propose a simple extension of the classic susceptible–immune–susceptible model to explore this phenomenon. The model is parametrized using RSV antibody data derived from healthy adults in Aotearoa, New Zealand. We consider a case study based on the prolonged stringent public health measures during the border closure years of 2020–2022 and compare these findings to observed hospitalization trends in Aotearoa, New Zealand. Our model predicts that diseases with very fast waning immunity are less likely to see increased infection rates after prolonged periods of stringent public health measures. However, diseases that wane moderately fast, such as RSV, are more likely to see a strong resurgence of cases when restrictions ease. Our results can be used to predict disease characteristics most likely to lead to strong resurgences after periods of prolonged restrictions and thus inform future public health responses.

## 1. Introduction

The restrictions associated with the COVID-19 pandemic during 2020−2022 saw multiple countries reporting decreased incidence rates of many, but not all, respiratory transmitted infections [1]. These nadirs were often followed by significant increases after non-pharmaceutical measures were lifted [2,3]. In many jurisdictions, hospitalization rates due to severe acute respiratory illnesses (SARI), for example influenza and respiratory syncytial virus (RSV), rose to very high levels in comparison with pre-pandemic levels [4]. Many theories have been proposed to explain the changes in epidemiology, including an ‘immunity gap’ [5]. This refers to a decline in population-level immunity caused by reduced pathogen exposure such as that which occurred during the COVID-19 pandemic due to non-pharmaceutical interventions including border closures, lockdowns and mask wearing.

Understanding the drivers of post-pandemic surges of globally important pathogens is critical to planning for future pandemics. Knowledge of which agents are more likely to show surges can help plan pre-emptive vaccination campaigns and/or prepare for hospital admission surges.

The classic susceptibility–infection–response (SIR) models of disease do not include varying levels of immunity. So they alone are not suitable for exploring the impact of reduced immunity. Although these models have been extended to partial differential equation (PDE) models to include varying susceptibility through the time since infection [6,7], they are primarily used to study vaccine responses, for example in tuberculosis [8] or COVID-19 [9]. The mechanisms of immunity gaps have also been posited through a qualitative model [10] and the cyclic behaviour of a susceptible–immune–susceptible (SIS) model [11].

Here, we propose a PDE model of waning immunity with strong similarities to the SARS-CoV-2 vaccination model of El Khalifi & Britton [9]. However, our aim is not to explore the effect of waning immunity after vaccination. Instead, our primary interest is in understanding the infection surges seen after prolonged periods of stringent public health measures (SPHMs). This is particularly relevant for illnesses that are potentially preventable with strategic vaccine campaigns, for example RSV, or those for which no vaccines are available, but health authorities would benefit from advance warning for severe hospital pressure. Our choice of model is justified and parametrized using time-series data on individual antibody levels derived from blood donors with RSV infection events in Aotearoa, New Zealand (NZ). The model is then utilized to simulate the effects of border restrictions and SPHMs on illnesses with different characteristics.

## 2. Data

Directly quantifying protective immunity remains challenging, particularly at a population level. As a result, measurable immune parameters known as correlates of protection are commonly used. Among these, antibody titres—especially neutralizing antibodies—are not only strongly associated with protection but are also mechanistically involved in preventing infection and disease [12–14]. Thus, antibody levels serve as both practical and immunologically meaningful proxies for modelling changes in population-level immunity.

Antibody time-series data collected from blood donors in Auckland, NZ, between March 2020 and March 2023 were utilized [15]. This comprised 150 donors selected to match ethnicity and gender demographics as per census data. During the pandemic years, Auckland was under varying levels of restrictions to reduce the spread of COVID-19, which concurrently reduced the spread of other respiratory illnesses. However, a brief border opening with Australia in 2021 introduced RSV, resulting in a short but intense wave of infection at the time [4].

We identified donors with significant antibody responses to RSV pre-fusion F protein (pre-F), the major RSV glycoprotein. Each donor’s antibody measurements were scaled individually by their baseline measurement, defined as the mean average of their three lowest antibody measurements. This scaling normalizes the responses allowing for comparison of the antigen-specific responses across donors. The peak response for each donor is their maximum measurement and time data were scaled so that the peak antibody response was on day zero. To meet inclusion criteria for an RSV pre-F antibody response, the donor must have the following three criteria:

(1) A peak unscaled antigen response in the top 25% of all recorded levels (greater than 5461.75 MFI) and a twofold increase over the donor’s baseline. This reduced the dataset to 29 donors.
(2) At least three antibody measurements both before and after the peak response measurement. This ensures the captured response was not in the middle of a decline or just starting to increase. This reduced the dataset to nine donors.
(3) A peak response for RSV that does not correspond with a similarly defined peak response to antigens from another pathogen investigated in the original study [15]. This removes possible co-infections and reduced the dataset to five donors.

**Figure 1.**
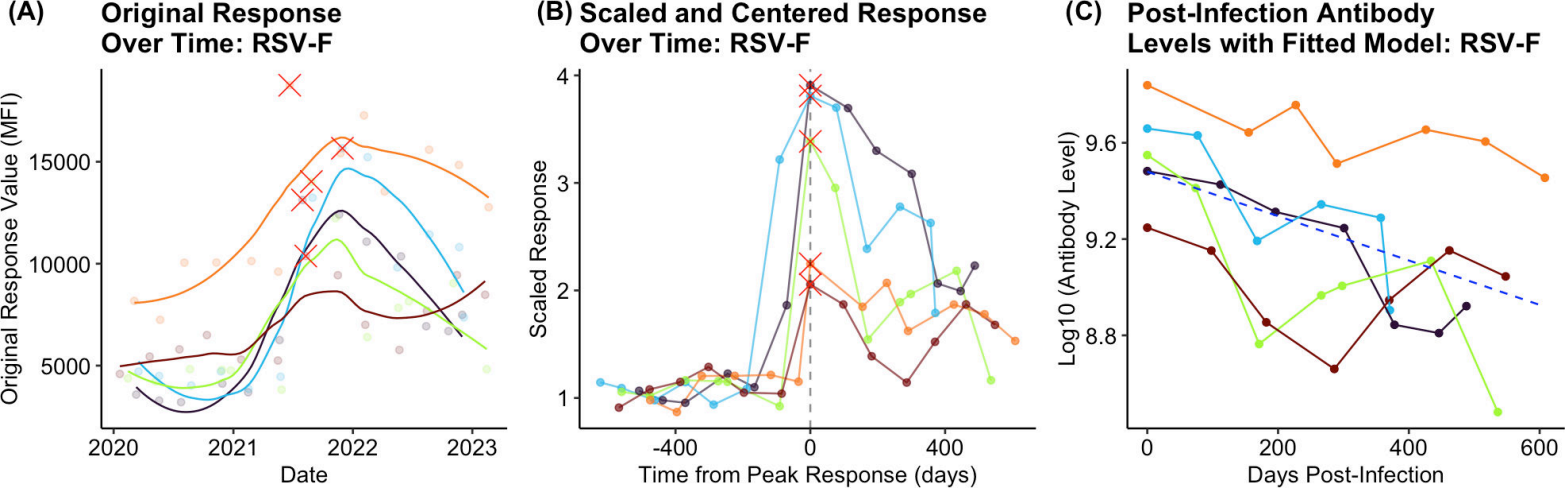
Time series showing the RSV-F antibody responses for the five individuals who showed a response. (A) Antibody responses on the original scale. Dots, data points; lines, line represents a locally weighted smoothing (locally estimated scatterplot smoothing, LOESS) curve for each donor, capturing the general trend of antibody responses over time without assuming a specific parametric model. The red cross marks the peak response. (B) Data are scaled by each individual’s baseline antibody level and the date of infection with the red cross marking the peak response. (C) Logged data post-infection only. Dots, data points; blue dashed line, fitted model.

This relatively small number of donors with peak RSV antibody responses that occurred in 2021/2022 (figure 1A) aligns with local epidemiology in both the overall prevalence of infections and the timing—that is, partial trans-Tasman border relaxations in 2021 and staged re-opening in 2022 but an absence of RSV in the population prior to these events [4]. Figure 1B shows the scaled time series for RSV antibodies in the five individuals defined as having an infection, while figure 1C shows post-infection data only.

We calculated the immunity waning rate as the time decay coefficient of a linear mixed effect model on the logged antibody response at and after the peak response with individual as a random effect:


log⁡(Response) ∼days after peak+(1|individual).

Using a logged response resulted in a model with a lower AIC and BIC value when compared to using an unlogged variable. The decay rate, r, is the coefficient of the time variable and the half-life of the response, $T_{50}=\ln\left(2\right)/r$, is the time taken for the expected antibody level to halve. The estimated decay rate was $-9.22\times 10^{-4}$ (p<0.001) giving an estimated half-life of 752 (95% CI 528–1324) days (calculated using the profile likelihood method: lmerTest package in R). Figure 1B shows the logged post-response time series and fitted model response for each donor.

As each donor’s time series of measurements starts with the peak response, they will, by definition, show a decline in antibody levels. To check that our results were not an artefact of this, we repeated the regression excluding the peak response at day zero. This decreased the estimated decay rate slightly to $-7.53\times 10^{-4}$ (p<0.01), giving an estimated half-life of 919 (95% CI 543–3254) days.

## 3. Model

Our model is very similar to that of El Khalifi & Britton [9], but a key difference is that the need for both recovered and susceptible classes is removed. Prior models often assume that infecteds move to a recovered class with partial immunity that wanes with time and finally into a susceptible class with no immunity. However, our data analysis showed exponentially decaying antibody levels occurring very quickly after infection, providing rationale for these classes to be combined through the use of a single infection waning function. We also extend our model to include both migration and seasonal infection rates. These extensions allow exploration of the phenomenon of immunity gaps in a level of detail not done previously.

We start with the classic SIS model of infectious diseases, where individuals are infected and then return to the susceptible population. $S\left(t\right)$ is the proportion of the population which is susceptible at time t, measured in days, and I(t) is the proportion of the population which is infected, i.e. S+I=1. We also allow immigration of infected individuals into the system at rate α and assume that the susceptible individuals leave the population at the same rate:


$$\frac{\text{d}S}{\text{d}t}=-\beta IS+\gamma I,$$

$$\frac{\text{d}I}{\text{d}t}=\beta IS-\gamma I.$$

An infected individual has a mean recovery time of 1/γ days and will infect β individuals each day in a fully susceptible population. The threshold for an epidemic is $R_{0}=\beta /\gamma$. If $R_{0}>1$, there will be a long-term equilibrium where 1-γ/β are infected, otherwise the disease will die out.

**Figure 2.**
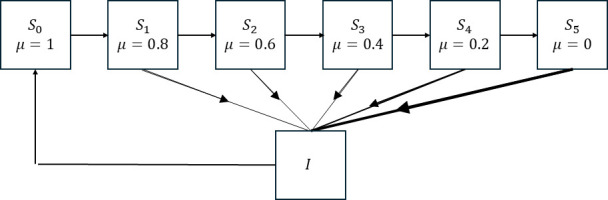
Immunity fades with time and is boosted by infection. Schematic of the immunity model for six immunity classes.


We extend this model to make the number of susceptibles a function of both time, t, and immunity, μ∈(0,1) (figure 2). We split the susceptible population into N+1 classes evenly distributed with spacing Δμ=1/N across a spectrum of susceptibility levels. Individuals in class $S_{i}$ have susceptibility $\mu_{i}=1-i/N$. An individual with μ=1 is fully immune and cannot catch the disease, an individual with μ=0 is fully susceptible. $S_{i}(t)$ is the proportion of the population with immunity $\mu_{i}$ at time t. We assume that the probability of catching the disease is $1-\mu_{i}$. Immunity wanes with time following the function $\rho (\mu_{i})$. Using these assumptions extends the single equation for $S\left(t\right)$ into N+1 equations:

$$\frac{\text{d}S_{i}}{\text{d}t}=\frac{-\rho \left(\mu_{i}\right)S_{i}+\rho \left(\mu_{i+1}\right)S_{i+1}}{\Delta \mu }-\beta IS_{i}\left(1-\mu_{i}\right)\;\text{for}\;i=1\ldots N-1.$$

The two boundary equations at μ=0 and μ=1 are slightly different. When an individual’s immunity has dropped to zero, they simply stay in that class and have probability 1 of catching the disease if they meet an infected person:


$$\frac{\text{d}S_{0}}{\text{d}t}=\frac{\rho \left(\mu_{1}\right)S_{1}}{\Delta \mu }-\beta IS_{0}.$$

The other end of the immunity spectrum, $S_{N}(t)$ and μ=1, is where recently infected individuals rejoin the susceptible population:


$$\frac{\text{d}S_{N}}{\text{d}t}=\frac{-\rho \left(\mu_{N}\right)S_{N}}{\Delta \mu }+\gamma I.$$

The equation for infecteds is now


$$\frac{dI}{dt}=\beta I\sum_{i=0}^{N}S_{i}\left(1-\mu_{i}\right)-\gamma I.$$

More complex compartment models have been put forward, but recently, at least, these have usually been in the context of vaccine-induced immunity, particularly to COVID-19, for example Vattiato *et al.* [16].

### 3.1. Continuous model

An obvious extension to the model is to consider immunity, μ, as a continuous rather than a discrete variable. Rather than having numerous classes of susceptible, $S_{i}(t)$, we have a single variable S(μ,t) where μ∈(0,1). In this case, our system of equations for the susceptible population becomes a single PDE:


$$\frac{\partial S}{\partial t}=\frac{\partial \left(\rho \left(\mu \right)S\right)}{\partial \mu }-\beta IS\left(1-\mu \right)$$

and the ODE for the infected population becomes


$$\frac{\text{d}I}{\text{d}t}=\beta I\int_{0}^{1}S\left(1-\mu \right)\text{d}\mu -\gamma I.$$

Infecteds who have recovered rejoin the susceptible population through the inhomogeneous boundary condition


$$\frac{\text{d}S\left(1,t\right)}{\text{d}t}=\gamma I.$$

This is a well-posed, though (almost certainly) analytically intractable, Cauchy problem. If it is solved numerically with finite differences and an upwind differencing scheme for the susceptibles PDE that discretizes the PDE into N equations and then uses numerical integration for the infected ODE, the resulting set of equations is identical to the system of ODEs presented initially.

### 3.2. Waning immunity

In the absence of an infected population, our hyperbolic PDE becomes


$$\frac{\partial S}{\partial t}=\frac{\partial \left(\rho \left(\mu \right)S\right)}{\partial \mu }.$$

Although the full PDE (most likely) cannot be solved, this simplified version does have a solution: waves with speed dictated by the function ρ(μ). We consider the initial condition of a Dirac delta function at μ=1, $S\left(\mu ,0\right)=\delta (\mu -1)$. This represents a fully immune population and allows us to understand how immunity wanes in the absence of re-infection. Lorenz *et al*. [15] showed that during the 2021 COVID lockdowns in Aotearoa, NZ, a period with strong social distancing and no external sources of infection, levels of antibodies for a number of common diseases showed exponential decay. If we choose $\rho \left(\mu \right)=-r\mu$, then the solution for our PDE is


$$S=\delta \left(\mu e^{rt}-1\right)e^{rt}=\delta \left(\mu -e^{-rt}\right).$$

The position of the Dirac function at time t, i.e. when $\mu -e^{-rt}=0$, is the immunity level of the population. So the immunity level of our cohort drops exponentially as required. As an example, the time taken to reach 50% immunity is $T_{50}=\ln\left(2\right)/r$, so if immunity falls to 50% after one year then r≈0.002; if immunity takes 10 years to fall to 50% then r≈0.0002.

### 3.3. Model behaviour

**Figure 3.**
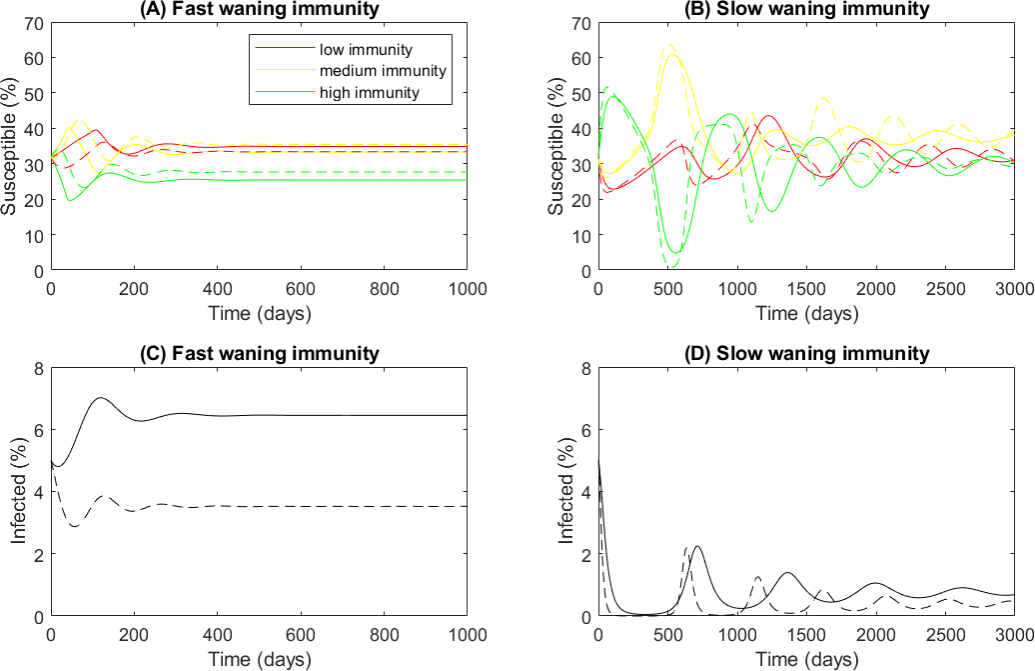
If immunity lasts longer, the disease takes longer to reach the steady state and the long-term number of infecteds is lower. Time series of the model solution with (A*,*C) fast $\left(r=0.01,\;T_{50}\approx 70\;\text{days}\right)$ and (B,D) slow ($r=0.001,\;T_{50}\approx 2$ years) waning immunity. Solid lines are: γ=0.1, β=0.2, i.e. $R_{0}=2$. Dashed lines are: γ=0.2, β=0.4, i.e. $R_{0}=2$. Red lines: low immunity, μ<1/3; yellow lines: medium immunity, 1/3≤μ<2/3; green lines: high immunity, μ≥2/3.

Figure 3 shows the model solution at two sets of parameter values. Our data analysis for RSV showed a wide range of estimates of the immunity waning speed, so the model was then examined at a wide range of parameter values and split parameter space into broad ranges to capture the full set of behaviours it could exhibit across a variety of infections. In the first set (figure 3A,C), immunity is very short-lived, r=0.01, i.e. $T_{50}\approx 70$ days, and solutions quickly converge to the long-term equilibrium where the disease is endemic. In the second set (figure 3B,D), immunity wanes more slowly, r=0.001, i.e. $T_{50}\approx 2$ years, and the solution oscillates for a much longer period as it converges. The long-term equilibrium has far fewer infected individuals when immunity is longer lived. The figure groups the susceptible population into three classes: high immunity (μ>2/3), medium immunity (1/3≤μ<2/3) and low immunity (μ<1/3). The initial condition is uniformly distributed immunity across the population, which has 500 immunity classes, and 5% of individuals are infected initially. Parameter descriptions and values (if not otherwise stated) are given in the nomenclature (table 1).

**Table 1.** Nomenclature, including model variables, parameters and initial conditions, descriptions and values (when fixed).

parameter	description	value
β	infection rate	d^−1^ (varies)
γ	recovery rate	0.1 d^−1^
$R_{0}$	epidemic threshold (definition from the classic SIS model)	$R_{0}=\beta /\gamma$
ρ(μ)	immunity waning function	ρ(μ)=−rμ
r	immunity waning rate	d^−1^
$T_{50}$	immunity half-life (time to reach 50% immunity)	$T_{50}=\ln\left(2\right)/r\;\left(days\right)$
λ	migration rate	d^−1^
κ	von Mises distribution parameter for seasonal infection rate	0.5
**variables**		
μ	immunity level	0 …1
t	time	days
I(t)	fraction of the population infectious at time t	
**initial conditions**		
I(0)	infecteds	0.05
S(μ,0)	susceptibles, uniformly distributed	$\left(1-I_{0}\right)U\left(0,1\right)$

In figure 3, both sets of results are for $R_{0}=2$ (using the definition of $R_{0}=\beta /\gamma$ from the classic SIS model) but the solid lines are γ=0.1,β=0.2, i.e. a disease with an average infectious period of 10 days. The dashed lines show γ=0.2,β=0.4, i.e. a disease with an average infectious period of 5 days. In the simple SIS model, this change would have made no difference to the final steady state. Now with waning immunity the faster acting disease reaches a lower steady state than the slow acting disease. Although the model transients can oscillate, in this simple version, around the parameter values of interest, we did not see any evidence of long-term stable oscillations like those seen when an SIS model effectively includes a delaying term, e.g. Munro & House [11] using an SIRS ODE model with a reduced immunity stage.

**Figure 4.**
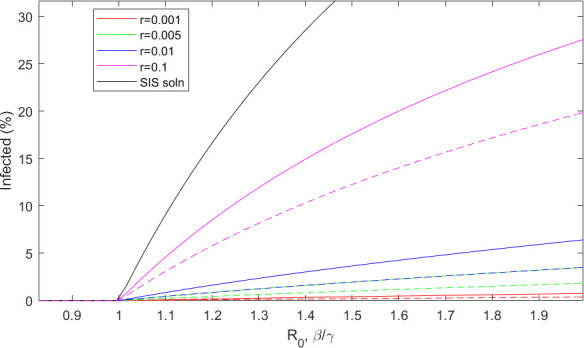
Immunity decay affects the long-term number of infecteds but not the threshold $R_{0}$. The equilibrium number of infecteds for a range of $R_{0}=\beta /\gamma$, i.e. as given in the simple SIS model, and immunity decay rates. Solid lines: recovery rate γ=0.1; dashed lines: recovery rate γ=0.2. As immunity decays faster, the solution tends to the traditional SIS model.

Figure 4 shows the long-term number of infecteds for a range of infection rates, β, at different immunity decay rates. Similar to the results of El Khalifi & Britton [9], the threshold parameter $R_{0}=\beta /\gamma >1$, derived from the standard SIS model, still determines whether the disease will die out or not, regardless of the immunity decay rate. Proving this for the PDE model is not straightforward but appendix A shows why $R_{0}$ for the SIS model is an upper bound for this model. The definition of $R_{0}$ as the threshold parameter for rising infection numbers is the number of infections caused by a single infected individual in a fully susceptible population. In a standard SIS model, if $R_{0}=\beta /\gamma >1$, the disease will become endemic and persist for all time; if $R_{0}<1$, the disease will die out after an initial infection wave has passed through the population. In our waning immunity model, we must consider the effective $R_{0}$, which is an interplay between the infection rate and the level of immunity of the population. After a wave of infection has passed through the population, the effective $R_{0}$ will be very low as many individuals are immune. As immunity wanes, the effective $R_{0}$ will increase, and ultimately, all individuals will be fully susceptible once again. In a continuous model, infection can never truly reach zero, so when the effective $R_{0}$ is greater than 1, there will be a rise in infections. This leads to the same threshold seen in the simple SIS model. We have not proved this rigorously, but the same conclusion was reached for the very similar model of El Khalifi & Britton [9].

Although the epidemic threshold follows the simple SIS prediction, the size of the endemic infected population does not. Now, it is dependent on both the immunity decay rate and the individual disease parameters, β and γ separately. Diseases with longer infectious periods and longer lasting immunity will have fewer infecteds at equilibrium. As the immunity decay rate increases and immunity decays quicker, the long-term equilibrium tends to the SIS steady state.

### 3.4. Migration

We extend the model to include infecteds entering the population at a constant rate λ. To simplify this, we assume that, as infecteds arrive, the same number of susceptibles leave, keeping the total population constant. We also assume that the immunity profile of those leaving matches the immunity profile of the population. In reality, both infected and susceptible individuals would be expected to arrive and leave. However, without good knowledge of the immunity profile of the arriving susceptibles, this model is a good approximation. Using the more succinct PDE notation:


$$\frac{\partial S}{\partial t}=\frac{\partial \left(\rho \left(\mu \right)S\right)}{\partial \mu }-\beta IS\left(1-\mu \right)-\lambda S,$$

$$\frac{\text{d}I}{\text{d}t}=\beta I\int_{0}^{1}S\left(1-\mu \right)\text{d}\mu -\gamma I+\lambda \int_{0}^{1}S\text{d}\mu$$

and


$$\frac{\text{d}S\left(1,t\right)}{\text{d}t}=\gamma I.$$

**Figure 5.**
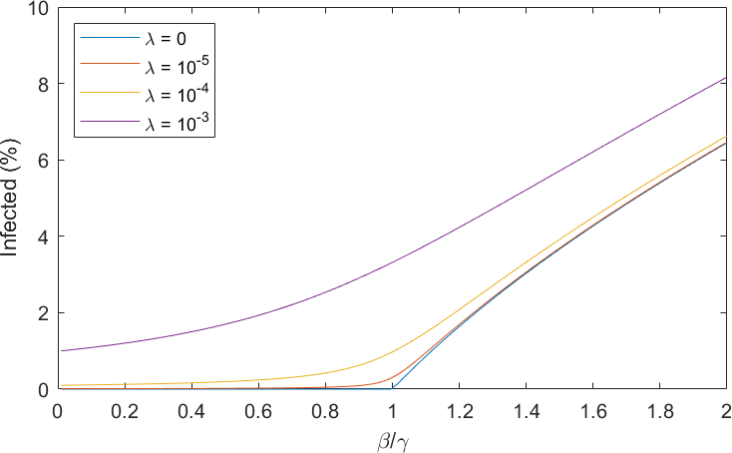
As infected individuals join the population through migration, the long-term number of infecteds increases. The steady state number of infecteds for a range of infection rates, β, and migration rates, λ. In the case of no migration (λ=0), then $\beta /\gamma =R_{0}$ in the standard SIS model. Other parameters are as in table 1.

Care must be taken in the choice of ρ(μ) as some choices could lead to immunity levels less than zero, which are not realistic. Figure 5 shows the long-term number of infecteds for a range of infection rates and migration rates. As expected, having a small number of infecteds arriving every day increases the long-term number of infecteds in the population. Even if $R_{0}$ is below the threshold predicted by the model without immigration, there will still be some infected individuals.

### 3.5. Time-dependent infection rates

A common trait of many illnesses is a seasonal infection rate. Winter flu season is challenging for many hospital systems as infection rates, and subsequent hospital admissions, rise significantly. We have extended our model to include a seasonal infection rate by making $\beta =\beta^{*}\theta (t)$. We let θ(t) follow a von Mises, or circular normal distribution that repeats every 365 days with κ, analogous to the variance, equal to 0.5. The peak infection rate is now $\beta^{*}$ and the minimum infection rate is approximately $0.4\beta^{*}$. This change effectively lowers the overall infection rate.

**Figure 6.**
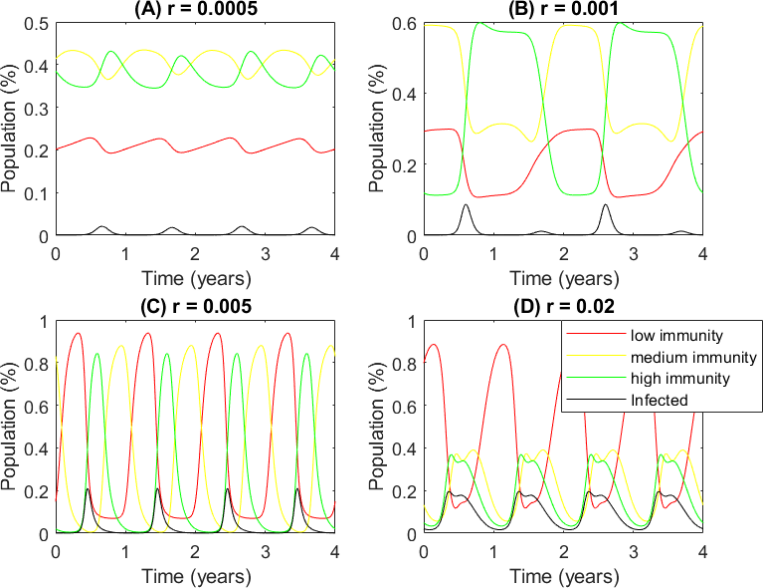
With seasonal infection rates, different rates of immunity waning lead to qualitatively different solutions. Time series of the system solutions at four rates of immunity waning with seasonal infection rates. (A) Slow waning (i.e. long lasting) immunity; (B) medium waning immunity; (C) fast waning immunity; (D) very fast waning immunity. Black lines: proportion of the population infected; red lines: low immunity, μ<1/3; yellow lines: medium immunity, 1/3≤μ<2/3; green lines: high immunity, μ≥2/3. Parameters: $\gamma =0.1,\;\beta^{*}=0.3,\;\lambda =10^{-5}$.

Figure 6 shows the solution of the system (after 50 years to ignore transient behaviour) for different rates of immunity waning. When immunity is slow waning and long-lived (figure 6A: $r=0.0005,T_{50}\approx 4$ years), the number of infecteds each season is very low. For moderate rates of immunity waning (figure 6B: $r=0.001,T_{50}\approx 2$ years) biennial solutions are seen cycling between alternate years of high and low infection. After a year of high infections, increased levels of residual immunity in the population have increased the effective $R_{0}$ and the following season shows a much lower infection rate. Unfortunately, after this low year, the residual immunity levels are reduced, and the following year, the effective $R_{0}$ is high once again with a correspondingly high infection rate, leading to the biennial behaviour seen here.

As immunity waning increases and shorter lived, this biennial behaviour vanishes to be replaced by an annual but larger infection peak (figure 6C: $r=0.005,T_{50}\approx 4$ months). When immunity wanes very quickly, the immunity curve for each season is flatter and, in some cases, with a double peak. Now, individuals infected very early in the season lose immunity so quickly (figure 6D: $r=0.02,T_{50}\approx 1$ month) that they can be reinfected in the same season. In this case, the longer infection season results in the total number of individuals infected (the area under the black curve) being higher despite the lower peak.

**Figure 7.**
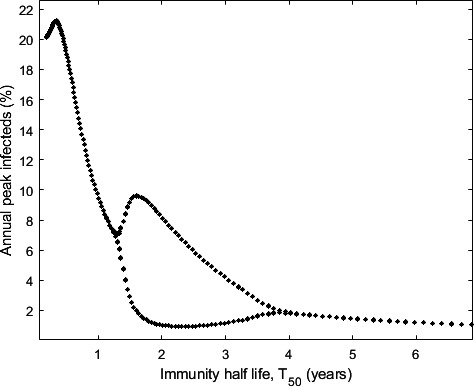
If immunity drops to 50% in 1−4 years, the system shows biennial infection peaks. Annual infected peak for a range of immunity waning rates, $T_{50}=\ln(2)/r$. Parameters: $\gamma =0.1,\;\beta^{*}=0.3,\;\lambda =10^{-5}$.

Figure 7 shows the annual peak immunity for a range of immunity waning rates. If the immunity half-life is between approximately 1 and 4 years, the model shows biennial peak infection patterns. There have been some claims of biennial infection patterns, e.g. RSV infections in children [17], while other diseases, e.g. *Mycoplasma* pneumonia [18,19], have longer cycles up to 4 years between peaks. With a seasonal forcing term, cycles with a period greater than a year are a feature of this model. However, individual heterogeneity and environmental noise would likely make them less regular.

## 4. Case study: COVID-19 lockdowns, March 2020–September 2022

During the pandemic, a period covering almost three winter illness seasons, NZ had stringent border controls in place and imposed strict public health measures, including lockdowns, to eliminate SARS-CoV-2 transmission. International borders were closed, with very limited entry via two weeks of managed isolation in place for two years. There were some brief exceptions for Australian citizens via a ‘trans-Tasman bubble’ in 2021. An alert level system was developed, and during alert levels 1 and 2, the least severe but longest duration restriction levels, the reduction in SARS-CoV-2 infection rate was estimated at 70% [20].

The first year of restrictions (2020) saw a sharp drop in RSV cases as the virus was effectively eliminated for 12 months [4]. In 2021, when the trans-Tasman bubble was briefly in place, RSV with an identical genotype to that circulating in Australia emerged in NZ with a very sharp and high peak incidence [21]. Similarly, out of season peaks were observed in other countries as restrictions eased, and often attributed to immunity gaps [22]. During the lockdown period, NZ also saw a significant drop in many other respiratory infections, which then rebounded dramatically after the borders were opened [4]. For example, large surges in influenza were observed after the pandemic years in NZ. However, not all respiratory pathogens followed these trends, and border restrictions did not affect cases of rhinovirus, human parainfluenza virus type 1 and enterovirus [4]. These different epidemiological patterns can be explored using our model.

**Figure 8.**
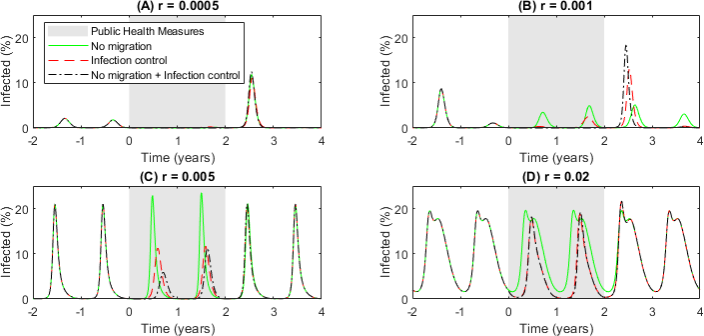
Diseases with slower rates of immunity waning are more prone to large infection peaks post-lockdown measures. Time series of the system solutions at four rates of immunity waning with seasonal infection rates during a two-year period of either no migration (solid green lines), SPHMs reducing infection rates by 30% (dashed red lines) or both (dot-dash black lines). (A) Slow waning (i.e. long lasting) immunity; (B) medium waning immunity; (C) fast waning immunity; (D) very fast waning immunity. Parameters: $\gamma =0.1,\beta^{*}=0.3$ and $\lambda =10^{-5}$ outside the period of public health measures; during the public health measures period γ=0.1 and (green solid line) $\beta^{*}=0.3$ and λ=0; (red dashed line) $\beta^{*}=0.21$ and $\lambda =10^{-5}$; (black dot-dash line) $\beta^{*}=0.21$ and λ=0.

Immunity gaps have been proposed as a driver of post-lockdown resurgences. Our model is an ideal testing ground for this theory and can help qualify the effect of SPHMs and border closures on infection levels due to immunity loss as well as the size of the rebound when these measures are lifted. Our model was set to run to steady state (100 years) with γ=0.1, and $\beta^{*}=0.3$, i.e. in the simple SIS model peak $R_{0}=3$, and low levels of immigration, $\lambda =10^{-5}$, and then for a period of two years we reduce either the peak infection rate, $\beta^{*}$, by 30%, to $\beta^{*}=0.21$ or the immigration rate to λ=0 or both $\beta^{*}$ and λ. After two years of simulated lockdown, the infection rate and immigration have returned to their pre-lockdown levels. Figure 8 shows time series for different rates of immunity waning.

When immunity wanes slowly (figure 8A: $r=0.0005,T_{50}\approx 4$ years) and infection rates are low, there is no annual infection peak if either migration is removed or infection rates are lowered. The combination of long-lasting immunity and reduced infection rates is enough to reduce the effective $R_{0}$ below 1 for the two years of lockdown. However, after these measures are removed, the lower immunity levels in the community have increased the effective $R_{0}$ considerably and result in a large peak in the first-year post-lockdown. For the next few years (not shown in the figure), the size of the peak oscillates until it reaches equilibrium again.

When immunity wanes a little faster (figure 8B: $r=0.001,T_{50}\approx 2$ years), the solutions are in the region of biennial cycles, although this has little impact on the overall behaviour. Now, border control measures alone (figure 8B, green line) are not enough to stop the infection curves during the two lockdown years, though the size of the peaks is much lower. When immigration is reintroduced after lockdown, the population immunity is enough to stop any significant surge in infections, and ultimately, the biennial cycles return. In the case of reduced infection rates but still some immigration (figure 8B, red line), the fairly long-lasting immunity is enough to make the first infection peak, initiated by migration and the seasonal rise in infection rates, during the lockdown period almost zero. The second peak, when some population immunity has been lost, is still much lower than usual. However, when the infection rate rises after the lockdown period, the population has markedly reduced immunity levels and a large resurgence of cases is seen in the first year of full infection rates. This is more significant in the case of no immigration and reduced infections (figure 8B, black line).

For very fast rates of immunity waning (figure 8C: $r=0.005,T_{50}\approx 4$ months; and figure 8D: $r=0.01,T_{50}\approx 1$ month), the effects of the lockdown period are much smaller. Both cases see reduced infection levels during the lockdown period, but these drops are small enough that there is no significant increase in infections the year after.

These simulations show the results for a peak infection rate of $\beta^{*}/\gamma =3.$ For diseases with a higher infection rate (and hence a higher peak $R_{0}$), similar patterns are observed—slower immunity waning results in larger post-lockdown peaks.

## 5. Discussion

Our model of seasonal infection rates with waning immunity is grounded in the well-established observation that antibody levels begin to decline soon after infection. Neutralizing antibodies, widely used as correlates of protection in viral infections such as SARS-CoV-2 and RSV, often follow an exponential decay trajectory over time [12,14]. We, therefore, propose that this decline in antibodies corresponds to a proportional reduction in protective immunity, an approach supported by both immunological evidence and recent modelling studies [14]. The model has many similarities with the classic SIS model, including, in the case with no migration, the threshold parameter $R_{0}$, which predicts whether infections will rise. A major difference from the classic models is the size of the endemic population, with this depending not only on $R_{0}$ (as in the classic model) but also on the individual parameters. Thus, if two diseases have the same $R_{0}$, the one caused by a pathogen with the longer infection period will be associated with a lower number of infected individuals in the long term. Our model also provides the rationale for biennial infection cycles, i.e. alternating high and low infection numbers, in ranges that correspond to parameters derived from data. Extending the model to include waning vaccinations, or even age classes, is relatively straightforward and could enhance the model’s real-world capability.

We have used the model to examine the recently posited immunity gap theory, whereby long periods of SPHMs resulting in low infection rates and lower immunity levels in a population, in turn, lead to high infection rates when the public health measures are removed [11]. Although we have presented our model results through a case study based on RSV infections during the COVID lockdowns of 2020−2022 in Aotearoa, NZ , our results are applicable elsewhere. Many countries saw post-lockdown resurgences of a range of illnesses. Although these would be represented by different parameter values in the model, the behaviours would be similar to those shown here. Even within the RSV data presented, there is a large uncertainty in the predicted immunity waning rate. Our approach of exploring a range of parameter values shows that the model contains only a small range of qualitative behaviours. In general, diseases where immunity wanes relatively slowly (in our example, β/γ=3 and immunity half-life is between 2 and 4 years) are more likely to have decreased infection rates during periods of SPHMs and then show a large resurgence when these measures are stopped. Diseases with a much shorter immunity half-life (e.g. less than a year) are more likely to continue during SPHMs, albeit with fewer infections, and show little change when the measures are dropped. These general conclusions hold for other circumstances, e.g. changes in infection rates due to different disease characteristics or less effective SPHMs during lockdown or higher rates of immigration during lockdowns.

Our model predictions are in good agreement with observed resurgences. Previous estimates of waning rates for RSV immunity are of the order $T_{50}\approx 200$ days, i.e. r=0.003 [23] with $R_{0}\approx 3$ [24]. Our own data (with a much smaller sample size and only including adults) give a similar estimate ($T_{50}\approx 500$ days (528–1324), i.e. r=0.0099). Our model predicts that this waning rate is in the region of parameter space where border controls and SPHMs are likely to significantly reduce infections, followed by a significant resurgence once these are released, and this aligns with local epidemiological data [4]. With recent vaccine developments for RSV, this modelling suggests that targeted vaccination campaigns during prolonged periods of SPHMs would be warranted. Conversely, immunity to pathogens such as rhinovirus and enterovirus appears to be shorter lived [25,26] and our model would predict that infection rates for these pathogens would remain largely unchanged after lockdown, as was observed in hospital admissions and lab data in NZ [4].

In conclusion, our model predicts that the immunity waning rate for a particular pathogen influences whether infection surges for the pathogen will be observed after prolonged periods of SPHMs. It highlights that diseases with moderately fast immunity waning are more likely to see a strong resurgence when restrictions ease. These pathogen characteristics and predictions can be used to inform future public health responses.

## Data Availability

The data in this paper have been used previously [15]. Due to privacy agreements with the data provider (NZ Blood Service) we are not able to make the data public at this time. The code that recreates the modelling analysis in the paper has been uploaded as electronic supplementary material [27].

## Appendix A

In the simple SIS model, the differntial equation for the number of infecteds is given by

$$\frac{\text{d}I}{\text{d}t}=I\left(\beta S-\gamma \right).$$

If the disease dies out or not depends on whether the number of infecteds increases or decreases. As I>0 this depends on the sign of

βS−γ.

In the simple SIS model, the basic reproduction number, namely the expected number of people an infected person will infect during their infectious period if the population is fully susceptible, i.e. S≈1, is given by

$$R_{0,sis}=\frac{\beta }{\gamma }.$$

If $R_{0,sis}<1$ the disease-free equilibrium is stable and the disease will die out.

For our model, it is not straightforward to calculate the basic reproduction. Instead, we show that $R_{0,sis}$ is an upper bound for the effective basic reproduction number of our model, $R_{0,eff}$.

We start with the differential equation for the number of infecteds:

$$\frac{\text{d}I}{\text{d}t}=I\left(\beta \int_{0}^{1}S\left(\mu \right)\left(1-\mu \right)\text{d}\mu -\gamma \right).$$

We know that S and $\left(1-\mu \right)$ are bounded functions of μ in $\left(0,1\right)$. Hence, by the mean value theorem of integrals, there exists $\mu_{c}\in \left(0,1\right)$ such that

$$\int_{0}^{1}S\left(1-\mu \right)\text{d}\mu =S\left(\mu_{c}\right)\left(1-\mu_{c}\right).$$

Our differential equation now becomes

$$\frac{\text{d}I}{\text{d}t}=I\left(\beta S\left(\mu_{c}\right)\left(1-\mu_{c}\right)-\gamma \right)$$

and we define our effective reproduction number as

$$R_{0,eff}=\frac{\beta S\left(\mu_{c}\right)\left(1-\mu_{c}\right)}{\gamma }.$$

The basic reproduction number for our model is always smaller than the basic reproduction number for the SIS model.
